# The role of lipid-based nano delivery systems on oral bioavailability enhancement of fenofibrate, a BCS II drug: comparison with fast-release formulations

**DOI:** 10.1186/s12951-014-0039-3

**Published:** 2014-09-24

**Authors:** Tengfei Weng, Jianping Qi, Yi Lu, Kai Wang, Zhiqiang Tian, Kaili Hu, Zongning Yin, Wei Wu

**Affiliations:** School of Pharmacy, Key Laboratory of Smart Drug Delivery of Ministry of Education, Fudan University, Shanghai, 201203 PR China; West China School of Pharmacy, Sichuan University, Chengdu, Sichuan 610041 PR China; Murad Research Center for Modernized Chinese Medicine, Shanghai University of Traditional Chinese Medicine, Shanghai, 201203 PR China

**Keywords:** Fenofibrate, Solid dispersion, Nanostructured lipid carrier, Self-microemulsifying drug delivery system, Bioavailability

## Abstract

The aim of this study was to compare various formulations solid dispersion pellets (SDP), nanostructured lipid carriers (NLCs) and a self-microemulsifying drug delivery system (SMEDDS) generally accepted to be the most efficient drug delivery systems for BCS II drugs using fenofibrate (FNB) as a model drug. The size and morphology of NLCs and SMEDDS was characterized by dynamic light scattering (DLS) and transmission electron microscopy (TEM). Their release behaviors were investigated in medium with or without pancreatic lipase. The oral bioavailability of the various formulations was compared in beagle dogs using commercial Lipanthyl® capsules (micronized formulation) as a reference. The release of FNB from SDP was much faster than that from NLCs and SMEDDS in medium without lipase, whereas the release rate from NLCs and SMEDDS was increased after adding pancreatic lipase into the release medium. However, NLCs and SMEDDS increased the bioavailability of FNB to 705.11% and 809.10%, respectively, in comparison with Lipanthyl® capsules, although the relative bioavailability of FNB was only 366.05% after administration of SDPs. Thus, lipid-based drug delivery systems (such as NLCs and SMEDDS) may have more advantages than immediate release systems (such as SDPs and Lipanthyl® capsules).

## Background

According to the definition of the Biopharmaceutics Classification System (BCS) proposed by Amidon in 1995, both BCS II and IV drugs are poorly soluble in aqueous solution [1]. About 40% of new drug candidates identified by chemical screening are poorly soluble in water (BCS II or IV drugs), which greatly hinders their translation into the clinic [2]. However, the transmembrane permeation behavior of BCS II drugs is significantly different to that of BCS IV drugs. Generally, the apparent permeability coefficient (*Papp*) of BCS II drugs is greater than 10^-6^, whereas the *Papp* of BCS IV drugs is lower than 10^-8^ owing to various barriers such as low dissolution rate, low transmembrane permeability, efflux by transporter in the gut wall and first pass effect by metabolic enzymes [3]. To improve the oral bioavailability of these drugs, novel formulation technologies or drug delivery systems have emerged, including solid dispersion [4,5], nanocrystals [6], cyclodextrin inclusion [7,8], nanoemulsions [9], polymeric and lipidic nanoparticles (e.g. PLGA nanoparticles, solid lipid nanoparticles and nanostructure lipid carriers) [10–12]. These formulations can enhance oral absorption of drug molecules by improving dissolution in the gastrointenstinal tract (GIT) [6], facilitating adhesive interactions within the mucosa [13], increasing drug stability and improving lymphatic transport [14]. Nevertheless, different formulations have distinguishing features and facilitate absorption by distinct mechanisms. Solid dispersion and cyclodextrin inclusion improve the dissolution rate of poorly soluble drugs, but do not increase transmembrane permeability [15,16]. Nanocrystals are a fast-release system that has similar effects to those of solid dispersion and cyclodextrin inclusion [6], whereas nanoparticles can alter the permeability of the intestinal membrane by uptake of intact nanoparticles, facilitating adhesion and retention in the GIT and improving membrane fluidity, thus leading to increased absorption via the paracellular or transcellular route [17,18]. Furthermore, the fate of nanoparticles containing lipids in the GIT is different to to that of polymer nanoparticles. Digestion products of lipid nanoparticles can solubilize lipophilic drugs and the presence of endogenous bile salts may alter the intrinsic permeability of the intestinal membrane [19,20]. Although each drug delivery system may be recognized to improve oral bioavailability of poorly soluble drugs, we aimed to identify the optimal formulation technology for delivery of BCS II or IV drugs. Therefore, in this study, we first compared the bioavailability of different drug delivery systems loaded with the BCS II drug, fenofibrate (FNB).

FNB, a widely used hypolipidemic agent, is a typical BCS II drug. Due to its very low solubility in aqueous solution, the oral bioavailability is limited by slow dissolution [21]. In the clinic, micronized FNB (Lipanthyl® capsules) showed significantly improved dissolution and enhanced oral bioavailability. More recently, various oral carrier systems were developed to increase oral absorption of FNB, including solid dispersion [4], a self-microemulsifying drug delivery system [22], liposome containing bile salts [23], mesoporous carbon [24], nanocystals [21] and lipid-based formulations [25]. Although these systems successfully increase the oral bioavailability of FNB, the optimal formulation remains to be identified by comparing the oral bioavailability of FNB after administration of different formulations.

Herein, the oral bioavailability of FNB-loaded into the lipid-based delivery systems, SMEDDS and NLCs, was compared with that of fast-release FNB SDPs and micronized Lipanthyl® capsules in beagle dogs.

## Results and discussion

### Preparation and characterization of SDP, NLCs and SMEDDS

**Figure 1:**
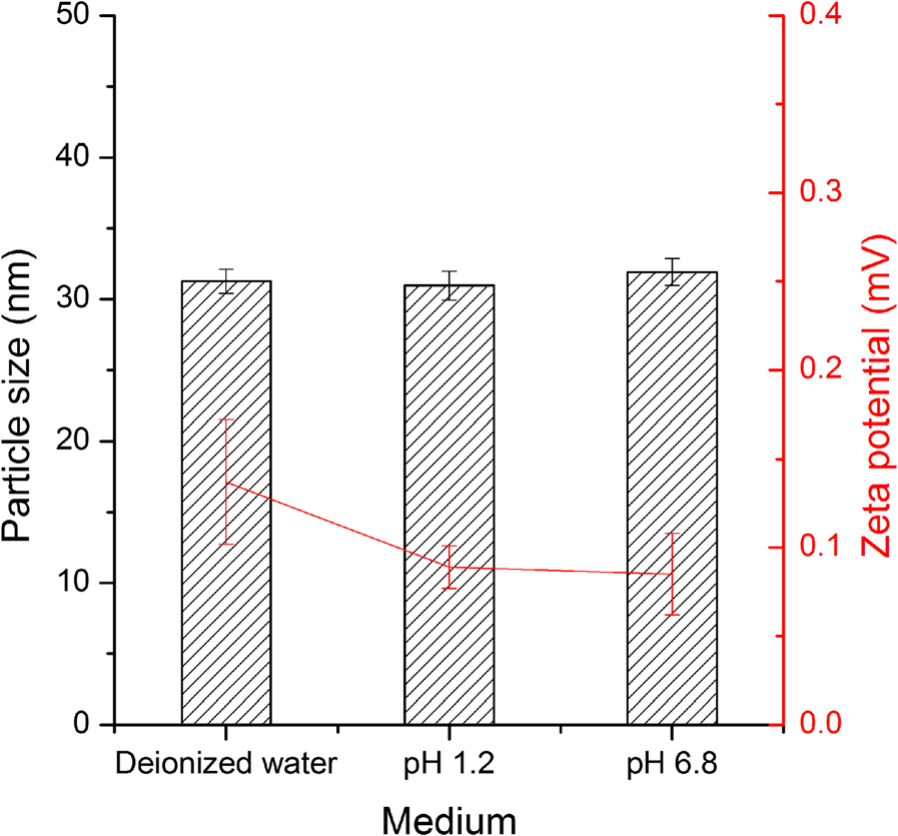
**The particle sizes and polydispersity index of FNB-loaded SMEDDS in different dispersion media (n = 3).**

FNB-loaded SDP, NLCs and SMEDDS were prepared successfully. Since FNB-loaded SDPs and NLCs were prepared according to our previous study [25,26], the detailed characterization data are not shown in this report. The particle size of the obtained NLCs was 93.76 ± 1.25 nm (polydispersity index (PDI), 0.222 ± 0.014), the zeta potential was -29.1 ± 4.1 mV and the entrapment efficiency was approximately 96.66 ± 1.01%, which are similar to the values obtained in our previous study [25]. FNB-loaded SMEDDS were microemulsified in deionized water, pH 1.2 HCl solution and pH 6.8 PBS immediately; the particle size and PDI are shown in Figure 1.

### Morphology

**Figure 2:**
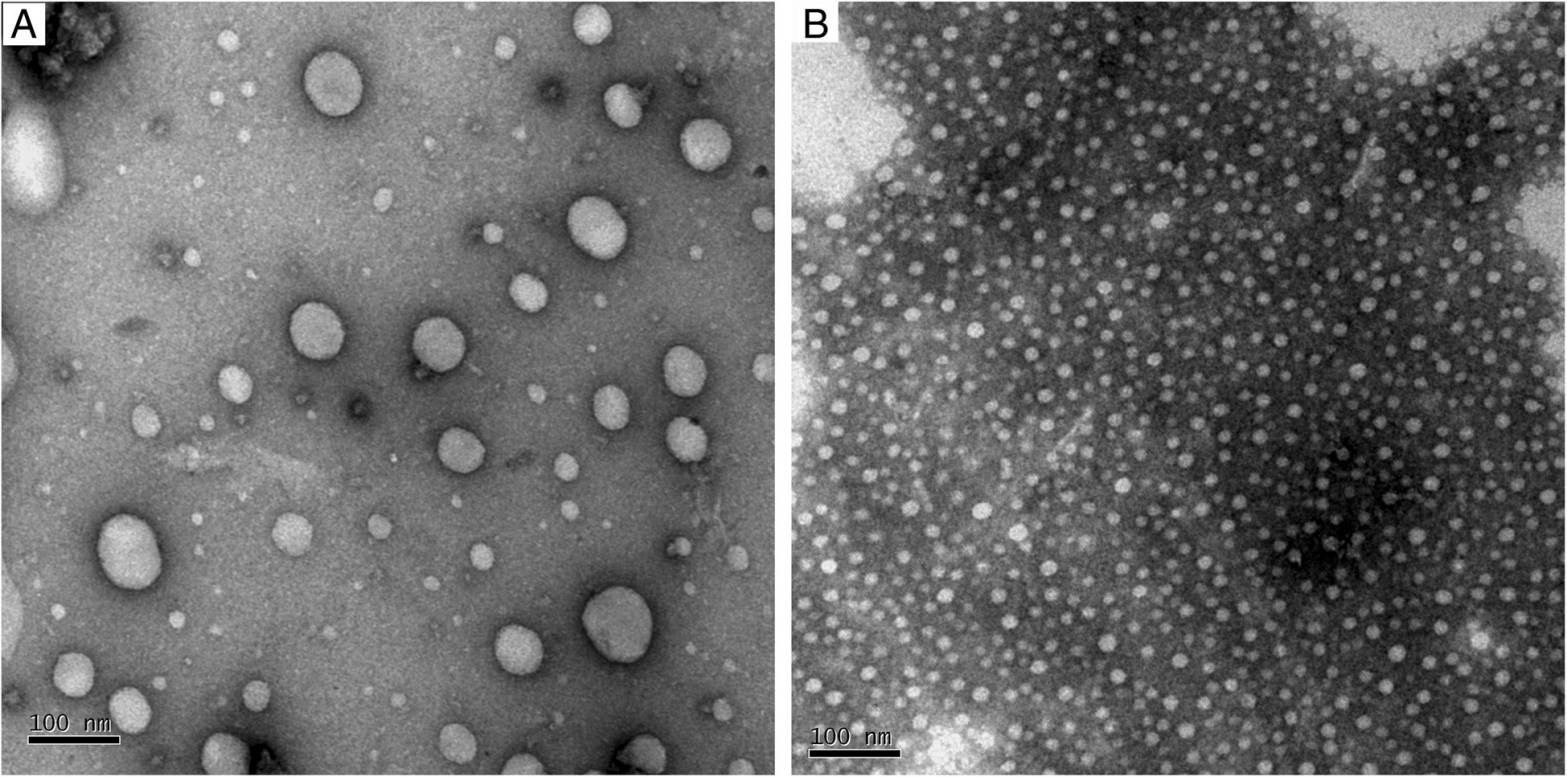
**Morphology of FNB-loaded NLCs (A) and microemulsions droplets formed by SMEDDS (B) observed by transmission electron microscopy.**

Transmission electron microscopy (TEM) was employed to observe the morphology of NLCs and microemulsions formed by SMEDDS; micrographs are presented in Figure 2. The NLCs (Figure 2A) were spherical in shape and approximately 100 nm in size. TEM showed that the SMEDDS were emulsified in deionized water to generate uniform spherical microemulsion droplets approximately 20 nm in size (Figure 2B).

### *In vitro* release

**Figure 3:**
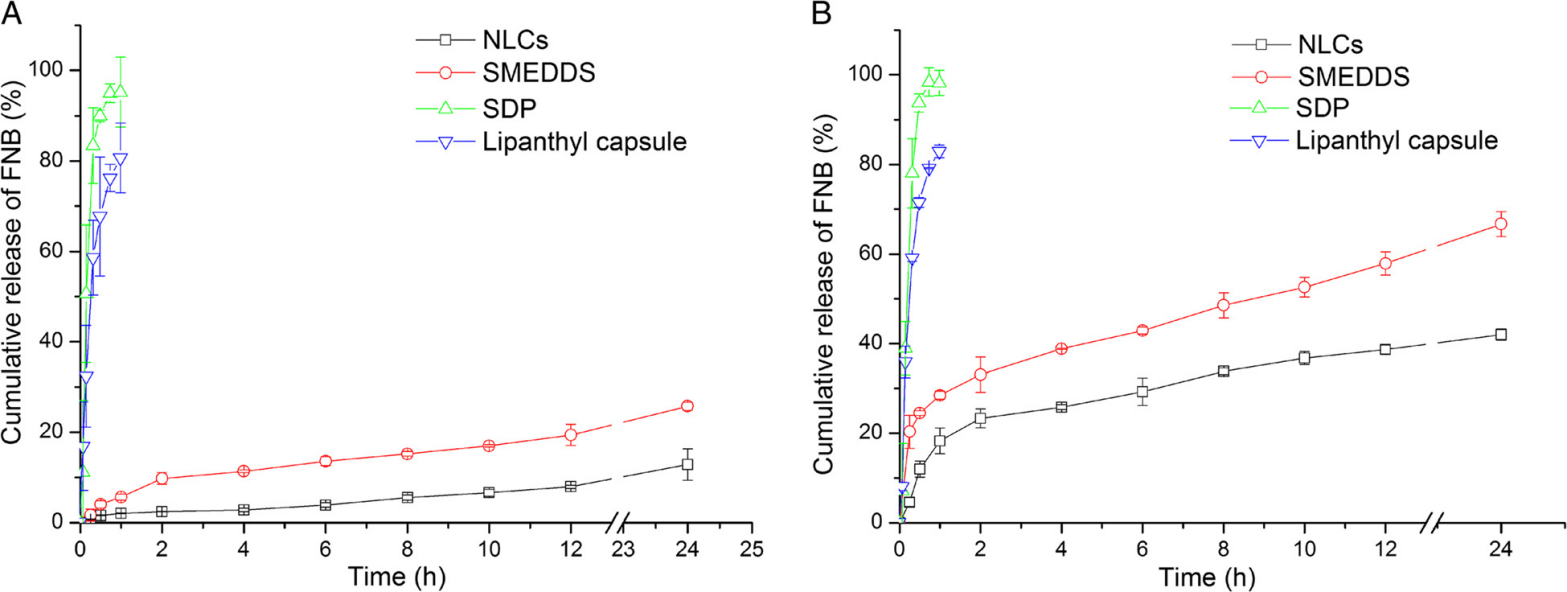
***In vitro*** **release profiles of FNB from SDP, NLCs, SMEDDS and Lipanthyl** ^**®**^ **capsules in release media without (A) or with (B) pancreatic lipase (n = 3).**

The profiles of FNB release from the three test formulations and the reference (commercial Lipanthyl® capsules containing micronized FNB) in the media with or without pancreatic lipase are shown in Figure 3. FNB was released rapidly from Lipanthyl® capsules and SDPs, with a cumulative release of more than 80% within 60 min. Only about 25% of the FNB was released from SMEDDS within 24 h and even less (about 12%) from the NLCs. However, pancreatic lipase changed the dissolution behavior of SMEDDS and NLCs. In release medium containing pancreatic lipase, the release of FNB from SDPs and Lipanthyl® capsules was not altered significantly compared with that in medium without lipase. Nevertheless, the release from SMEDDS and NLCs was evidently improved, with more than 60% and 40% of FNB released from SMEDDS and NLCs in 24 h, respectively. The similarity factor (*f*_*2*_), which is recommended by FDA for evaluation of the similarity of release profiles [27], was employed to evaluate the influence of lipase on FNB release according to the following formula:

$$ {f}_2=50\times \lg \left(\sqrt{\left[1+\left(\frac{1}{\mathrm{n}}\right){\displaystyle \sum_{i=1}^n}{\left({x}_{ti}-{x}_{ri}\right)}^2\right]}\times 100\right) $$

where *x*_*ti*_ and *x*_*ri*_ are the cumulative release of time interval “*i*” in release medium with and without pancreatic lipase, respectively, and n is the time interval. When *f*_*2*_ is between 50 and 100, the variation in every observation point between the two release profiles is not more than 10%, which is considered to represent similarity. If *f*_*2*_ < 50, the two release profiles are considered to be dissimilar. The *f*_*2*_ of the four formulations are displayed in Table 1. The release profiles of FNB from Lipanthyl® capsules and SDPs were not altered by the addition of lipase to the release medium, although the release profiles from SMEDDS and NLCs in two release media differed considerably, which indicates that intestinal lipase is important to the release of poorly soluble drugs from lipid-based drug delivery systems, such as SMEDDS and NLCs.

**Table 1:** **The** ***f***_***2***_ **values of release profiles of FNB in release media with or without pancreatic lipase**

**Formulations**	**Lipanthyl** **®** **capsules**	**SDP**	**SMEDDS**	**NLCs**
*f*_2_	67.1	60.7	36.5	42.4

### Oral bioavailability

**Figure 4:**
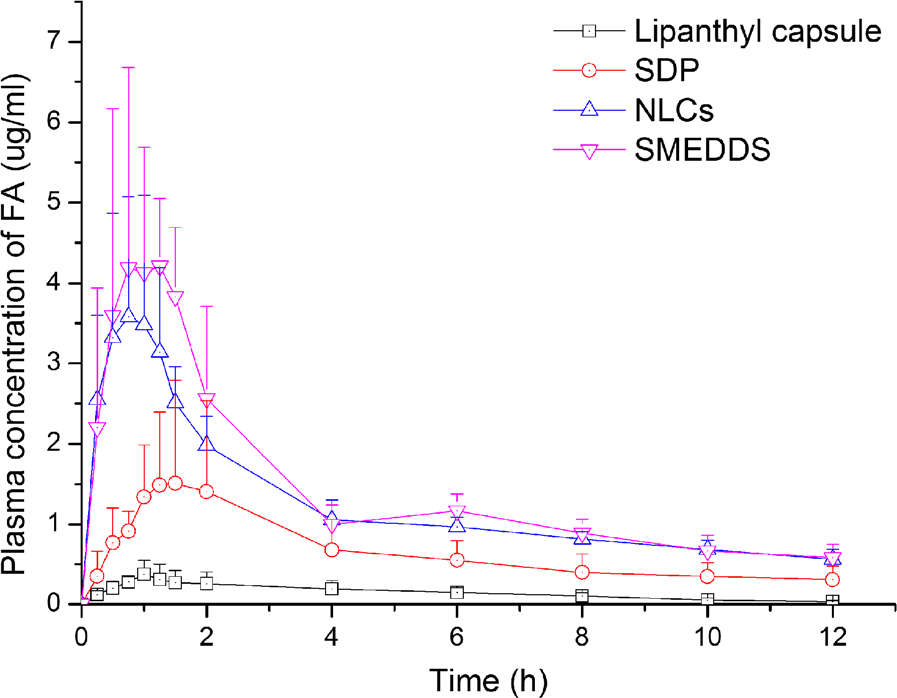
**Mean plasma concentration-time profiles of fenofibric acid in beagle dogs after oral administration of FNB-loaded SDP, NLCs, SMEDDS and Lipanthyl** **®** **capsules (n = 6).**

To illustrate the optimal formulation for BCS II drugs, oral bioavailability of FNB-loaded SDPs, NLCs and SMEDDS in beagle dogs were compared. The mean plasma FNB concentration versus time plots of the four formulations are shown in Figure 4 and the pharmacokinetic parameters obtained by analysis based on statistical moment theory are shown in Table 2.

**Table 2:** **The main pharmacokinetic parameters of fenofibric acid in beagle dogs after oral administration of FNB-loaded SDP, NLCs, SMEDDS and Lipanthyl® capsules (n = 6)**

**Parameters**	**Lipanthyl** **®** **capsules**	**SDP**	**NLCs**	**SMEDDS**
*C*_max_ (μg/mL)	0.41 ± 0.17	1.82 ± 1.18*	3.87 ± 1.40*^#^	5.31 ± 1.18*^#▲^
*T*_max_ (h)	1.04 ± 0.29	1.13 ± 0.59	0.92 ± 0.34	0.96 ± 0.43
*t*_1/2_ (h)	3.40 ± 1.55	9.58 ± 7.64	8.09 ± 3.25*	6.25 ± 2.15*
*MRT*_(0-t)_(h)	4.24 ± 0.24	4.45 ± 0.18	4.22 ± 0.42	4.00 ± 0.28
*AUC*_(0-t)_ (μg/mL*h)	2.13 ± 0.69	7.81 ± 4.36*	15.04 ± 2.34*^#^	17.26 ± 3.43*^#^
F_1_ (%)	—	366.05	705.11	809.10
F_2_ (%)	—	—	192.62	221.03
F_3_ (%)	—	—	—	114.75

F_1_, F_2_ and F_3_ are relative bioavailability of other formulations compared with Lipanthyl® capsules, SDP and NLCs, respectively.

*P < 0.05, compared with Lipanthyl® capsule.

^#^ *P* < 0.05, compared with SDP.

^▲^ *P* < 0.05, compared with NLCs.

After oral gavage administration of the three FNB formulations to beagle dogs, the C_max_ and AUC of all of the formulations were improved compared with those of Lipanthyl® capsules. NLCs and SMEDDS in particular exhibited enhanced absorption compared with SDPs. The relative bioavailability of NLCs and SMEDDS were 705.11% and 809.10%, respectively, compared with Lipan-thyl® capsules, while that of SDPs was only 366.05%. Compared with Lipanthyl® capsules, the T_max_, MRT and t_1/2_ of fenofibric acid showed no significant changes after oral administration of all three formulations.

Theoretically, the oral bioavailability of BCS II drugs is restricted mainly by poor dissolution in the GIT. Generally speaking, the oral bioavailability of BCS II drugs is improved greatly if the *in vitro* dissolution is enhanced [28]. Therefore, micronization, nanosuspension, solid dispersion and cyclodextrin inclusion are widely used to improve the oral bioavailability of BCS II drugs [28]. Previous *in vitro* and *in vivo* evaluations of the reference, Lipanthyl® capsules, which are a product of micronized FNB and nanosuspensions of FNB suggested the FNB is rapidly released from Lipanthyl® capsules, SDPs and nanosuspensions, and that SDPs or nanosuspensions improve the oral bioavailability of FNB compared with that of the Lipanthyl® capsules [29]. Similar dissolution does not lead to the same oral absorption, which may be due to the various influences of the GIT contents on dissolution of drugs from the different formulations.

Although FNB was released very slowly and in small amounts from lipid-based drug delivery systems, such as NLCs and SMEDDS, the cumulative release of FNB increased with introduction of lipase. Pancreatic lipase, bile salts and phospholipids are continuously secreted into the GIT. Lipid-based drug delivery systems are digested by pancreatic lipase to form secondary structures, such as mixed micelles, cubic or hexagonal nanoparticles and vesicular carriers [30]. Therefore, drugs can be solubilized in these secondary derivatives when lipid-based formulations are digested [31]. SDPs, nanosuspensions or micronized drugs significantly increase drug dissolution, but oral absorption is promoted only by the original absorption pathways of the drug itself. Nevertheless, lipid-based drug delivery systems may enhance the absorption of drugs through diverse pathways [32].

On the one hand, NLCs and SMEDDS can adhere to the gut wall to increase retention time in GIT. The particle sizes of NLCs and SMEDDS were below 100 nm, which endows them with a massive specific surface area and facilitates the adhesion of nanoparticles by the mucus layer [33,34]. On the other hand, many reports have suggested that the digestion of lipid-based drug delivery systems in the GIT is the most important factor required to enhance the absorption of poorly soluble drugs [35–37]. Exogenous lipids stimulate the secretion of biliary lipids (bile salts, phospholipids and cholesterol), which combine with lipid digestion products to generate a series of colloidal species, including micelles, mixed micelles, vesicles and emulsion droplets [38]. These colloidal species provide a reservoir of solubilized drug at the absorptive site and generate the concentration gradient required to drive improved absorption. Luminal amphiphiles, such as bile salts, may also enhance the solubilization of drugs by improving wetting at concentrations below the critical micellar concentration [39]. Thus, the drug concentration increases during the digestion process, which improves the transport across non-stirred water layers and then the bio-membranes. In addition, fatty acids and monoglycerides produced during digestion increase the fluidity and permeability of membranes due to their surface activity, which is also an important factor in enhancing drug absorption [40]. Furthermore, lymphatic transport can also increase the oral bioavailability of lipophilic drugs [41]. Lipid vehicles may enhance lymphatic transport of lipophilic compounds by simulating the production of chylomicrons. Lipophilic drugs enter the lymphatic system in association with the triglyceride core of the chylomicrons [42]. The digestibility of the vehicle is a prerequisite for the production of the fatty acids necessary to drive chylomicron production [43].

## Conclusion

FNB-loaded SDPs, NLCs and SMEDDS were prepared and their *in vitro* and *in vivo* properties were compared. SDPs significantly increased the release of FNB in medium with and without lipase, which is similar to the characteristics of Lipanthyl® capsules. Pancreatic lipase improved the release of FNB from NLCs and SMEDDS remarkably. However, the oral bioavailability of FNB after administration of NLCs and SMEDDS was significantly higher than that of SDP and Lipanthyl® capsules (*P* < 0.05). Therefore, lipid-based drug delivery systems (such as NLCs and SMEDDS) are more advantageous than the other drug delivery systems (solid dispersion or micronization) for BCS II drugs, due to the multiple absorption enhancement mechanisms. Lipid-based drug delivery systems may be an excellent candidate for oral formulation of insoluble drugs.

## Methods

### Materials

FNB was purchased from Nhwa Pharmaceutical Group (Xuzhou, China). Polyvinylpyrrolidone (PVP K30) was kindly gifted from China Division, ISP Chemicals Co. (Shanghai, China). Non-pareil pellets (Suglets® sugar spheres PF101, 710–850 μm in diameter) were provided by NP Pharm (Bazainville, France). Precirol ATO 5 and Captex 100 were kindly provided by Gattefossé Co. (Saint Priest, Cedex, France) and Abitec Co. (OH, USA), respectively. Polysorbate 80 (Tween-80) was supplied by Shenyu Pharmaceutical and Chemical Co., Ltd (Shanghai). Oleoyl macrogolglycerides (Labrafil M® 1944 CS) and diethylene glycol monoethyl ether (Transcutol P®) were purchased from Gattefossé Co. Ethoxylated castor oil (Cremophor® EL) was obtained from BASF Corporation (Ludwigshafen, Germany). HPLC grade methanol and acetonitrile were purchased from Tedia (Carson City, NV, USA). Deionized water was prepared using a Milli-Q purification system (Millipore, Billerica, MA, USA). All other chemicals were of analytical grade and were used as received.

### Preparation of FNB-loaded delivery systems

#### Preparation of solid dispersion pellets (SDP)

FNB-loaded SDPs were prepared using a Mini-Glatt fluid-bed coater (Wurster insert; Glatt GmbH, Binzen, Germany) based on previously established procedures [26]. FNB, PVP K30 and sodium dodecyl sulfate (SDS) (4:3:3, w/w) were dissolved in 90% ethanol. The resulting solution was sprayed through a nozzle (0.5 mm in diameter) onto the fluidized non-pareil pellets to obtain a coating weight gain of approximately 100%. The detailed operating conditions were as follows: product temperature, 35°C − 40°C; air flow rate, 97–103 m^3^/h; spray rate, 0.6 mL/minute; atomizing air pressure, 1.4–1.5 bar. The pellets were further dried for 15 min after coating completion.

#### Preparation of nanostructured lipid carriers (NLCs)

The NLC suspension was prepared by the melting-emulsification method according to our previously described procedures [25]. Briefly, 1.14 g solid lipid phase (Precirol ATO 5) and 0.48 g liquid lipid phase (Captex 100) were melted at 80°C and mixed. Then 60 mg FNB was dissolved in the lipid mixture. The melted mixture was then dispersed in a hot (80°C) aqueous solution (30 mL) containing Tween-80 (2%, w/v) for 3 min at a rate of 8,000 rpm using a high-speed Ultra Turrax blender (QilinBeier, Jiangsu, China) to produce the coarse emulsion. Subsequently, the coarse emulsion was homogenized using a high-pressure homogenizer (Microfluids, Nano DeBee, USA) for three cycles under 20,000 psi. The obtained hot NLC suspension was cooled to room temperature for use in further investigations.

#### Preparation of self-microemulsifying drug delivery systems (SMEDDS)

The formulation of FNB-loaded SMEDDS was performed according to previously described methods with modifications [44]. Briefly, FNB, Labrafil M 1944 CS (oil phase), Cremophore EL (surfactant) and Transcutol P (co-surfactant) were mixed in ratio of 40/520/585/195 (w/w). The obtained SMEDDS was stored at 4°C before use.

### Measurement of particle size

Particle size was measured by Zetasizer Nano® (Malvern Instruments, Malvern, UK) equipped with a 4 mW He-Ne laser (633 nm) at 25°C. The NLC suspension was diluted 15-fold with deionized water before measurement. The particle size of SMEDDS was determined after microemulsification in deionized water. Three measurements were conducted, and the number of runs in each measurement was automatically determined by the software.

### Transmission electron microscopy (TEM)

TEM was used to characterize the morphology of NLCs and SMEDDS. Prior to examination, microemulsion drops were obtained by emulsifying SMEDDS in deionized water. The NLCs suspension and microemulsion droplets were then placed on copper grids and negatively stained with 2% (w/v) phosphotungstic acid for 5 min at room temperature. Finally, the grids bearing NLCs and microemulsion droplets were observed with a JEM-1230 transmission electron microscope (JEOL, Tokyo, Japan).

### Entrapment efficiency of NLCs

The entrapment efficiency of NLCs was determined by ultrafiltration. Briefly, 0.4 mL NLCs was added to an ultrafiltration tube (100 kD) and then centrifuged for 10 min at 4,000 × *g*. The concentrations of FNB in the filtrate (*C*_*free*_) was determined by HPLC directly. The concentration of FNB original NLCs (*C*_*total*_) were determined as following method. Briefly, 0.4 mL of NLCs suspension was dissolved in 100 mL methanol. The FNB released into methanol from NLCs rapidly with the help of ultrasound. After ultrasound treatment of 20 min, the mixed solution was centrifuged for 10 min under 10,000 × g. The supernatant was injected into HPLC to determine *C*_*total*_. The entrapment efficiency (EE) was calculated according to the following equation.

$$ \mathrm{E}\mathrm{E}=\frac{C_{total}-{C}_{free}}{C_{total}}\times 100\% $$

### Release test

The release test was performed in a ZRS-8G dissolution tester (Tianda Tianfa Technology Co. Ltd, Tianjin, China) according to the Chinese Pharmacopoeia (2010) Appendix Method III. To clarify the effect of lipase on the release of lipid formulations (NLCs or SMEDDS), we selected two different release media; phosphate balanced saline (pH 6.8) containing 2% Cremophor EL with or without pancreatic lipase (100 IU/mL). Four formulations (containing 3 mg FNB) were added into 100 mL release medium that was thermostatically maintained at 37 ± 0.5°C and stirred at a revolution speed of 100 rpm. SDP was sealed into hard gelatin capsules. Samples of 0.5 mL were withdrawn at specific time intervals and immediately ultrafiltered (Millipore, 100 kD) at 4,000 × *g* for 10 min. The ultrafiltrate was assayed for FNB by HPLC as described later in the text.

### Bioavailability study

The bioavailability of SDPs, NLCs and SMEDDS containing FNB was evaluated in beagle dogs using commercially available Lipanthyl® capsules (micronized FNB, Solvay Pharma) as a reference. Beagle dogs (adult males, 15.0 ± 0.5 kg) used in the experiments received care in compliance with the Principles of Laboratory Animal Care and the Guide for the Care and Use of Laboratory Animals. Experiments followed protocols approved by the Fudan University Institutional Animal Care and Use Committee.

Four formulations were administered to the dogs by oral gavage at an equivalent dose of 3 mg/kg FNB. Blood samples (1.5 mL) were then collected into heparinized tubes at designated time intervals: 0.25, 0.5, 0.75, 1, 1.25, 1.5, 2, 4, 6, 8, 10 and 12 h. Plasma was separated by centrifugation for 10 min at 4,000 × *g* and frozen at -18°C for subsequent analysis. FNB, as a prodrug, is rapidly metabolized into its major active metabolite, fenofibric acid (FA), after absorption. Intact FNB cannot be detected in the plasma after oral administration; therefore, pharmacokinetic evaluation of FNB was based on the quantification of FA in the plasma [45]. FA in dog plasma was extracted by liquid-liquid extraction procedures established in our previous study and the concentration of FA was determined by HPLC [23].

Pharmacokinetic parameters were calculated by non-compartmental analysis based on statistical moment theory using DAS professional software version 2.0 (Anhui, China). The pharmacokinetic parameters, such as peak plasma concentration (*C*_max_), the time to maximum plasma concentration (*T*_max_), and the area under the concentration-time curve between 0 and 12 h (*AUC*_0-12_) were determined.

### HPLC analysis

Both *in vitro* and *in vivo* samples were determined by HPLC system (Agilent 1260 series, California, USA) comprising an auto sampler, a pump, a column oven, and a tunable ultraviolet detector. The analytical column was a C18 column (Diamonsil®, 5 μm, 4.6 mm × 250 mm, Dikma, China) guarded with a refillable precolumn (C18, 2.0 mm × 20 mm, Alltech, USA). The flow rate was 1.0 mL/min. The UV-detector was set at a wavelength of 287 nm. The column temperature was set to 40°C. In terms of *in vitro* determination of FNB, the mobile phase consisted of methanol and deionized water mixed at a ratio of 90/10 (v/v). However, the mobile phase was composed of a mixture of methanol, water and 10% phosphoric acid (70/30/1, v/v/v) for *in vivo* determination of FA. Indomethacin (10 μg/mL) was used as an internal standard [23].

### Statistical analysis

All data were expressed as mean ± standard deviation (SD). One-way ANOVA followed by Tukey’s test was performed to assess the statistical significance of differences. Results with *P* < 0.05 were considered statistically significant.
